# What drives the adoption of online health communities? An empirical study from patient-centric perspective

**DOI:** 10.1186/s12913-023-09469-6

**Published:** 2023-05-23

**Authors:** Qianyao Zhang, Runtong Zhang, Xinyi Lu, Xijing Zhang

**Affiliations:** 1 Department of Information Management, School of Economics and Management, Beijing Jiaotong University, Beijing, 100044 China; 2 School of Management and E-business, Zhejiang Gongshang University, Hangzhou, Zhejiang China

**Keywords:** Online health communities (OHCs), Information demands, Behavioral intention, eHealth literacy, Relation quality, Price value

## Abstract

**Background:**

Online health communities (OHCs) provide platforms for patients to seek advice from physicians and receive professional suggestions online. It can improve the efficiency of patients’ diagnosis of simple diseases and alleviate hospital congestion. However, few empirical studies have comprehensively explored the factors influencing patients’ intention to use OHCs through objective data. This study aims to fill this gap by identifying key factors that influence patients’ acceptance of OHCs and proposing effective ways to promote the applications of OHCs in China.

**Methods:**

Based on the Unified Theory of Acceptance and Usage of Technology (UTAUT), extended with additional constructs identified with patients’ information demands in OHCs, this study developed a research model and proposed nine hypotheses. An online survey involving 783 valid responses was conducted in China to collect data to validate the proposed model. Confirmatory factor analysis and partial least squares (PLS) path model were conducted for instrument validation and hypothesis testing.

**Results:**

Price value, eHealth literacy, and performance expectancy are the most prominent constructs in the study context. Interestingly, relation quality was also found to have a significant positive relationship with behavioral intention.

**Conclusions:**

Based on these findings, OHC operators need to create a user-friendly platform, improve information quality, set reasonable prices, and establish consummate security systems. Physicians and related organizations can raise awareness and assist patients in developing the skills to appropriately comprehend and utilize information in OHCs. This study contributes to both technology adoption theory and practice.

**Supplementary Information:**

The online version contains supplementary material available at 10.1186/s12913-023-09469-6.

## Introduction

As Internet technology advances, “Internet Plus” is widely integrated with various industries. Online health communities (OHCs) are developed with the application of Internet technology in the field of medical and health care [1, 2]. They connect patients, physicians, hospitals, and other medical ecosystems through the internet and specifically provide an information exchange platform for patients and physicians [3].

Encouraging patients to use online health communities (OHCs) in conjunction with offline treatments is necessary. OHCs provide patients with the convenience of seeking help and advice online, regardless of location and time [4, 5], and improve the possibility of timely diagnoses. Besides, during the period of the COVID-19 pandemic, OHCs can effectively reduce patients’ visits to hospitals, thus minimizing patient-to-patient and patient-to-physician physical contact and ensuring the safety and well-being of both patients and physicians. In addition, since the interactions between patients and physicians are one-to-one and not face-to-face, patients’ privacy can be protected [6, 7]. OHCs are also proven to play an important role in helping physicians optimize their time utilization [4, 8, 9], reduce healthcare costs [10], and alleviate issues related to hospital congestion and uneven distribution of healthcare resources [11]. Currently, many countries recognize intelligent medical services as an important project of public health development. For example, the Chinese government encourages strengthening the capacity of Internet medical services [12], illustrating the development of OHCs in line with current national strategies. Given the benefits of OHCs and the recent inevitable development trend, patients’ widespread use of OHCs is critical.

A comprehensive understanding of the factors determining patients’ acceptance of OHCs is a prerequisite for facilitating patients’ acceptance of OHCs [13], which is therefore necessary. Currently, OHCs are still not widely used [14, 15]. The lack of patient engagement can limit the success and sustainability of OHCs, undermine the development policies of many countries, and weaken health care’s modernization and rapid development [12]. It raises questions about the factors that influence patients’ decisions to use OHCs. Previous works mainly adopted or extended several famous technology adoption models based on the literature to examine predictors of OHC adoption [7, 13, 16–19]. Within these models, the Unified Theory of Acceptance and Use of Technology (UTAUT) model integrates the eight most widely used models [20] to measure the use intention of information technology [21, 22]. It can explain 70% of the variance of behavioral intention and better explain the influencing factors of behavioral intention than the eight models mentioned before [23, 24]. Given the advantages of the UTAUT, researchers often adopted the UTAUT model or used it as the basic theoretical framework to develop their research models to better reflect the characteristics of OHCs [25–27]. Although these studies enhanced the understanding of the adoption of OHCs, the variables do not fully explain the factors influencing patients’ adoption of OHCs. There are two reasons. First, for studies using UTAUT as the basic model, the variables included in UTAUT cannot fully explain patients ‘usage intention towards OHCs. Venkatesh et al. [20] and Alam et al. [13] have criticized UTAUT’s predictive power in healthcare technology adoption as unclear and insufficient, particularly in developing countries, and suggested the inclusion of context-specific determinants to improve its predictability [13, 28]. Second, for studies that extend the UTAUT model, factors other than the basic UTAUT model were selected from subjective judgments or developed theories, resulting in a lack of comprehensive coverage of all factors that impact patients’ acceptance of OHCs. For instance, Hoque and Sorwar [25] extended UTAUT with systematic variables (i.e., technology anxiety and resistance to change) based on their knowledge and literature. Sun et al. [27] developed the research model integrating UTAUT, credibility online health information, and perceived risk based on the literature. These previous studies ignored the impact of patients’ ability and relationships with the platforms, which would impact patients’ acceptance of OHCs. The limited perspective of the determinants of OHC adoption restricts our understanding of patient behavior and hinders the development of effective strategies to promote OHC uptake. Addressing this gap requires objective data to identify patients’ actual needs and extend the UTAUT model to fully explain the factors that determine patients’ intentions and behaviors in using OHCs.

The availability of health information through OHCs can significantly affect individuals’ health management and perception of using these platforms [29–31]. Consequently, an analysis of patients’ information demands in OHCs can reveal factors that influence patients’ adoption of OHCs. This study aims to explore patients’ online information demands in OHCs and to identify the critical factors that impact patients’ behavioral intentions and adoption of OHCs. Three research questions are proposed as follows:

**RQ1.** What are the information demands of patients in OHCs?

**RQ2.** Based on the information demands of patients in OHCs, what potential factors can be identified that influence patients’ adoption of OHCs?

**RQ3.** What factors determine patients’ adoption of OHCs?

The contributions of this study are as follows. First, this study presents a comprehensive conceptual model that fills the gaps in the literature on OHCs by rigorously clarifying the factors influencing patients’ adoption of OHCs and their impact. This study extended the UTAUT model based on the analysis results of patients’ information demands of OHCs. Patients’ information demands of OHCs can reflect the factors that patients in OHCs concern about, thus providing references for the selection of influencing factors of patients’ intention to adopt OHCs. Introducing influencing factors selected based on patients’ information demands in OHCs to the research model can expand the coverage of influencing factors on behavioral intention to use OHCs, strengthen the understanding of patients’ acceptance of using OHCs, and improve the explanatory capacity of the research model. Second, this study expands on the impact of relation quality and eHealth literacy in patients’ use of OHCs, enriches the literature on relation quality and eHealth literacy, and reveals the effects of patients’ competencies and relationships with the platform on the behavioral intention of OHCs. Third, this study identifies the key factors influencing patients’ behavioral intention of using OHCs and their impact mechanism. It validates the research model using data from patients in OHCs in China. These findings can inform decision-makers to facilitate the adoption of OHCs in China and other developing countries similar to China in terms of digital health technology development.

## Research model and hypotheses

### Patients’ information demands of OHCs

We gathered data from a prominent Chinese OHC named *Qiuyi*, with the largest accessible question-and-answer (Q&A) data. Q&A data refers to posts in OHCs that contain patient inquiries and physician responses. Patients’ inquiries depict the information they seek and can reflect their information demands. Employing data mining technology, we acquired the Q&A data from January 1, 2011, to December 31, 2021, totaling 1,202,732 Q&A data. Next, word segmentation, stop word deletion, TF-IDF algorithm, and Word2vec were conducted to process the data. We selected the top 148 words with the highest TF-IDF values, which were most pertinent to patients’ information demands (words after the top 148 TF-IDF values had a too-low frequency of occurrence). Then, a hybrid clustering method combining k-harmonic means and overlapping k-means algorithm (KHM-OKM) was used to cluster these words. This method was proven to be effective for medical texts [32]. Finally, the data were divided into ten groups. To increase the accuracy of the results, we also performed topic identification based on the Latent Dirichlet Allocation (LDA) method. Perplexity was used for the determination of the number of topics. When k = 10, the perplexity was at a minimum. Thus, the optimal topic number was ten [33], consistent with the previous method’s findings. We evaluated the clustering effect using the Sum of the Squared Errors (SSE), Silhouette Coefficient, and Davies-Bouldin Index (DBI). The results revealed that when the number of clusters was ten, all indicators were optimal (SSE had a clear turning point at k = 10; Maximum Silhouette Coefficient, equal to 0.32; Minimum DBI, equal to 0.03). These validation results demonstrated a good clustering effect [34–36]. Next, we invited five medical experts from different specialties to summarize and organize the topics’ meanings based on the words under each topic’s distribution and meaning [37]. In this process, the nominal scale categories are independent, mutually exclusive, and exhaustive, and each of the five experts operates independently [38]. The experts summarized the topics based on the relevant literature and the Unified Medical Language System (UMLS). Ultimately, patients’ information demands in OHCs were summarized as therapy, pathology & etiology, test, prevention, self-management, effect, risk, emotion, price, and reliability. To test the robustness of the results, we used Fleiss’ Kappa to measure the inter-rater agreement. The results revealed k = 0.81, indicating a high consistency of expert labeling [39].

In patients’ information demands of OHCs, the demands for therapy, pathology & etiology, and test reflect patients’ expectations of receiving effective assistance from OHCs, which are consistent with the concept of performance expectancy in the UTAUT model [20]. In addition, the information demand for risk reflects patients’ concern about potential risks, which can be measured by perceived risk [40]. Moreover, the demand for price reveals patients’ perception of the expenditure in OHCs, which can be measured by price value [41]. Additionally, the prevention demand and self-management demand show patients’ expectations to keep healthy by understanding the information in OHCs, which are applicable to be measured by eHealth literacy [42]. Finally, effect demand and reliability demand reveal that the degree of trust impacts patients’ behavioral intention to use OHCs, and the emotion demand manifests patients’ satisfaction level with OHCs. These demands can be measured by relation quality [43, 44].

### The UTAUT variables

#### Performance expectancy

Venkatesh et al. [20] defined performance expectancy as “the degree to which an individual believes that using the system will help him or her to attain gains in job performance” (pp. 447). Performance expectancy reflects users’ desire to maximize efficiency and productivity [45]. Users will be more inspired to use new technology if they think it will improve their lives [20, 46]. Several studies stated that performance expectancy is an essential factor that affects users’ intention to use the system. For example, through empirical research, Hoque & Sorwar [25] proved that performance expectancy is one of the important factors affecting users’ intention to use mHealth. Baishya & Samalia [47] found that performance expectancy significantly promotes users’ positive intention to use the system. In the context of OHCs, patients can be motivated to use OHCs if they believe that OHCs can provide them with effective medical services and accurate information. Therefore, the following hypothesis is proposed:

##### H1

Performance expectancy positively influences the behavioral intention to use OHCs.

#### Effort expectancy

Effort expectancy is defined by Venkatesh et al. [20] as “the degree of ease associated with the use of the system” (pp. 450). Users are concerned about how easy it is to use the new system and how much energy they need to invest in using the system [48]. They prefer to try to use simple and easy-to-use techniques. Effort expectancy has been identified by many studies as a critical factor that directly affects users’ behavior intention of using e-health systems, clinical decision support systems, and mobile health. For instance, Tavares et al. [49] pointed out that effort expectancy significantly influences users’ adoption of electronic health records. Rana et al. [50] stated that users’ acceptance of a system depends on their ability, such as how easy it is to use. The individual with higher effort expectancy believes that using the new system requires less effort, thereby, tends to use the system [20, 51]. OHCs are new systems for patients, so there is a significant correlation between their effort expectancy and their behavioral intention. Thus, we assume the following hypothesis:

##### H2

Effort expectancy positively influences the behavioral intention to use OHCs.

#### Social influence

Social influence represents the extent to which an individual is affected by the feelings, cognitions, and behaviors of surrounding groups [20]. It is usual for people to share their experiences of using new technologies with others. The thoughts of the surrounding people can impact an individual’s thinking. Thus, social influence has a substantial impact on an individual’s behavior. Encouragement and positive feedback from surrounding groups significantly motivate individuals to adopt technology [46]. Biasutti & Frate [52] reported that social influence is a prominent factor influencing users’ behavioral intention to use the technology. Alam et al. [13] studied the influencing factors of users’ usage behavior of mHealth services in Bangladesh. The results indicated that social influence positively affects users’ usage intention. Positive social recognition can increase patients’ trust in the OHCs and potentially improve their satisfaction, thus promoting their willingness to use OHCs. Therefore, we derive the following hypothesis:

##### H3

Social influence positively influences the behavioral intention to use OHCs.

#### Facilitating conditions

Facilitating conditions refer to the level of technical or organizational support an individual can obtain while using the system [20]. The conditions for promoting the development of new technology include help-desk support, professional support, and peer support [45]. The support can provide a solid foundation for positive feelings and usage behavior of the system. High levels of organizational support can promote positive beliefs about using health technology. According to Martin et al. [17], facilitating conditions positively influence usage behavior. Cimperman et al. [53] proved that facilitating conditions are the main factor in strengthening patients’ usage of telehealth services. Mengesha et al. [54] found that facilitating conditions are one of the most prominent constructs that affect telemedicine adoption. In the context of OHCs, patients will perceive OHCs as reliable and thus be more likely to use OHCs if they can get valid help when they have problems. Therefore, we propose the following hypothesis:

##### H4

Facilitating conditions positively influence the usage behavior of OHCs.

#### Behavioral intention

Behavioral intention refers to the extent to which an individual perceives his or her intention to use the service of mHealth [13]. Researches have shown that behavioral intention is the best predictor of actual use, including the use of technology in the health field [55–57]. Several studies found that an individual’s behavioral intention is the most crucial factor of his/her usage behavior for mobile medical technologies or services [20, 58, 59]. Therefore, we suggest that:

##### H5

Behavioral intention positively influences the usage behavior of OHCs.

### Perceived risk

Perceived risk is first proposed by Bauer [60], defined as the negative psychological expectations that individuals may have. It can be divided into two aspects: the uncertainty of an individual’s decision-making results and the severity of the consequences of wrong decisions [60]. In many studies, perceived risk in the context of the Internet includes psychological risk, social risk, financial risk, performance risk, time risk, and privacy risk [61, 62]. Due to the fear of taking risks, the threats perceived by users will increase their expectations of negative results and result in reduced intentions [63–65]. Yang et al. [62] argued that perceived risk negatively affects users’ behavioral intention. Lu et al. [40] and Lee et al. [66] also supported that by providing statistical evidence. If patients believe that using OHCs can ensure their property security and privacy, they will feel reassured to use OHCs, which can improve their intention of using OHCs. Therefore, we propose the following hypothesis:

#### H6

Perceived risk negatively influences the behavioral intention to use OHCs.

### Price value

Price value refers to the cognitive trade-off between the benefits individuals obtain from an application and its cost. It is one of the strongest factors that motivate people to continue using mobile Internet services [41]. Yuan et al. [67] confirmed that price value would positively influence behavioral intention, and Ali [68] provided evidence to certify that price value has a positive impact on use intention. During the period of using information technology services, people tend to compare the value they got with the price they paid [69]. Therefore, patients would increase their usage level if they think that the value they get is higher than the expenditure they paid for using information technology services [70]. Besides, patients expect to obtain higher-quality information or experience better service when spending more [71]. Thus, we propose the following hypothesis:

#### H7

Price value positively influences the behavioral intention to use OHCs.

### eHealth literacy

eHealth literacy refers to the ability to get and use the information to solve problems and enhance self-health care from OHCs [42]. People have been able to obtain medical services through the Internet within the modern health information environment, which is conducive to enhancing patients’ ability of self-care management [72]. Consequently, in addition to health awareness, the ability to understand health information is important for people [42, 73]. Norman & Skinner [42] stated that individuals’ eHealth literacy requires basic reading and writing skills, computer knowledge, a basic understanding of science, and comprehension of the social environment. eHealth literacy is capable of affecting patients’ attitudes toward the use of OHCs. People with high eHealth literacy are likely to use social media platforms to obtain health-related information [74, 75]. If patients cannot fully understand the health information in OHCs, they would underestimate the value of the information provided by OHCs, thereby reducing the usage level of OHCs [76, 77]. In contrast, if patients have strong abilities to discriminate the information, understand and apply the information appropriately, they can identify useless information and use the appropriate information to help themselves. In this case, they will obtain more valuable information than the patients with lower eHealth literacy and thus have more positive intentions to use OHCs. Therefore, the following hypothesis is postulated:

#### H8

eHealth literacy positively influences the behavioral intention to use OHCs.

### Relation quality

Relation quality refers to the outcome produced by the patients in interacting with OHCs, which is used to evaluate the relationship between the patients and OHCs [44]. It is a crucial determinant of behavioral intention, which will further affect the usage behavior of information technology platforms [78]. Relation quality incorporates patients’ usage satisfaction related to the OHCs and patients’ trust toward OHCs [44]. Specifically, satisfaction refers to users’ overall opinion and experience when using technology services. It is the most important driver of usage intention [79]. Trust can be further explained by the user’s perceived competence, reliability, and empathy for the product or service [80]. It is an important factor in avoiding uncertainties that would negatively influence experienced users’ intention to use information technology, thus increasing the continuous intention of users to use the technology [80, 81]. These variables positively impacted the technology platform’s continuance intentions [82]. For example, in accordance with Liébana-Cabanillas et al. [83], in the context of OHCs, if patients trust in hospitals, physicians, information, and services in OHCs, they will believe that OHCs are reliable and authoritative, resulting in a positive intention to use OHCs. Liébana-Cabanillas et al. [80] also stated that satisfaction could encourage users to adopt technology platforms. As the integration of satisfaction and trust, relation quality has also been proven to positively affect users’ continuance usage intention of information technology by Zhang et al. [44] ’s research. Therefore, we suggest the following hypothesis:

#### H9

Relation quality positively influences the behavioral intention to use OHCs.

### Research model

**Fig. 1:**
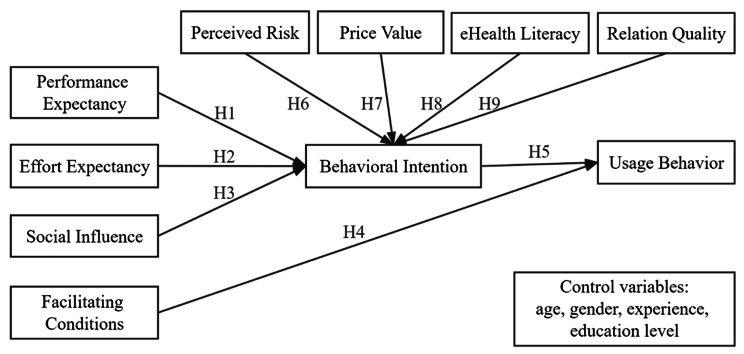
Theoretical research model

Based on the preceding hypotheses, we propose the following model, as illustrated in Fig. 1, for the outcome variable, usage behavior, by incorporating eight independent variables (i.e., performance expectancy, effort expectancy, social influence, facilitating conditions, perceived risk, price value, eHealth literacy, relation quality) and one mediator (i.e., behavioral intention). Age, gender, experience, and education level are taken into account in this study as control variables to identify the potential demographic effects.

## Materials and methods

### Instrument development

To ensure reliability and validity, well-known and published instruments have been adopted in our study [1, 84, 85]. The five-point Likert scale response format that ranges from 1 (strongly disagree) to 5 (strongly agree) was used to measure items. The ten construct variables associated with the research model were covered in the survey tool. Performance expectancy (5-item), effort expectancy (4-item), social influence (3-item), facilitating conditions (4-item), and behavioral intention (3-item) were adopted from Venkatesh’s study [20]. In addition, usage behavior (4-item) and perceived risk (4-item) were developed by the studies by Sun et al. [27]. Furthermore, price value (3-item) under the background of Internet health, eHealth literacy (8-item), and relation quality (5-item) were adopted from the study of Yuan et al. [67], the study of Norman and Skinner [73], and the study of Chen et al. [43], respectively.

### Analysis tool selection

In this study, descriptive statistics were used to describe the sample demographics. Confirmative factor analysis was applied to study the instrument’s construct validity, and Cronbach’s α was calculated to assess the internal reliability of the subscales. Structural Equation Modeling (SEM) is a linear statistical modeling technique used to analyze the correlation between variables in the model [84, 86]. The Partial Least Square (PLS) method is one of the statistical methods for SEM. It effectively explains the multi-collinearity between manifest and latent variables and the specification of structural model errors [87]. It can also evaluate the reliability and effectiveness of the structural model [87]. This study applied the partial least squares structural equation modeling (PLS-SEM) to test the research model formulated in the previous sections and evaluate the explanatory capacity of the model as well as the relationship between the constructions [55]. We used SmartPLS to analyze and assess the relationship among the variables in the research model.

### Data collection and respondent profile

This study was approved by the Ethical Committee of the School of Economics and Management, Beijing Jiaotong University. We carried out cross-sectional research in China. First, the scale was translated from English into Chinese. The translation process of the scale was based on previous studies [88, 89]. And the translated scale was validated by five experts to ensure that the translated scale was accurate, unambiguous, readable, and easy to understand. Then, to ensure the validity of the questionnaire, a pretest including fifty respondents was conducted by using the convenience sampling method. Based on the feedback from the pretested respondents, the questionnaire was revised and finalized with 43 items that best fit the constructs in the research model.

With the help of a medical association in China, we conducted formal surveys in different private and public hospitals in China. Given that we intended to study patients’ usage intention in the context of the OHCs environment, we forwarded questionnaires through WeChat (a widely-used mobile social application in China) to ensure that the participants use mobile applications. We set 7 RMB as a reward for all respondents’ time and efforts. The respondents were asked whether they had used OHCs before, and only those who had experience with OHCs participated in the interview. We used the convenience sampling method as the main survey instrument and used the judgment sampling method in some aspects.

Data were collected from February 2021 to October 2021. A total of 950 questionnaires were sent to the participants. Among them, 934 were received, with a response rate of 98.32%. The criteria of the invalid response included: (1) Responses with the same options for all the questions, (2) the response that included incomplete answers, and (3) the response that the completion time was less than the average time (we calculated the average completion time as 96s based on the pretest). With this criteria, 783 responses were valid. Therefore, the validity rate was 83.83% (783/934). According to Kass & Tinsley [90], the ratio of the number of scale items and the sample size should exceed 1:5. In the study, there were 43 items, so 783 was a good sample size. Table 1 reveals the demographics of the sample. There were 415 participants (53.00%) aged between 21 and 30, 481 participants (61.43%) were women, and 686 participants (87.61%) held at least a bachelor’s degree. Therefore, the participants were mainly young people, most of whom had obtained higher education, with a relatively high proportion of women. The target respondents of this study were participants who had used OHCs. Since OHCs are web-based platforms, the target respondents of this study were highly correlated with the users of web-based information platforms. Previous studies showed that users of web-based health information platforms were mostly young, female, and educated [91], which is consistent with the characteristics of our sample. Therefore, the samples were reasonably representative. The reliability and validity of the sample are discussed in Sect. 4.1.

**Table 1:** Sample demographics (N = 783)

Demographic Characteristics	N (%)
**Age**	
Under 20 years old	74 (9.45%)
21–30 years old	415 (53.00%)
31–40 years old	141 (18.01%)
41–50 years old	115 (14.69%)
Above 50 years old	38 (4.85%)
**Gender**	
Male	302 (38.57%)
Female	481 (61.43%)
**Education Level**	
Senior high school or lower	44 (5.62%)
Junior college	53 (6.77%)
Bachelor’s degree	514 (65.65%)
Master’s degree	151 (19.28%)
Doctor’s degree	21 (2.68%)
**Job**	
Employees in government offices and public institutions	101 (12.90%)
Professional and technical workers	168 (21.46%)
Factory workers	72 (9.20%)
Commercial service workers	103 (13.15%)
Retirees	17 (2.17%)
Students	287 (36.65%)
Liberal professionals	29 (3.70%)
Farmers	6 (0.77%)

## Results

### Reliability and validity of the instrument

According to the previous studies, the reliability and validity of the structural scales were suitable measurement instruments for evaluating the reliability and fit index of the research model [6, 84]. We used SmartPLS 3.3.2 software to test the reliability and validity of the measurement scales. The reliability of the instrument was assessed by Cronbach’s α, which was between 0.75 and 0.95 (Table 2), so the reliability was acceptable (Cronbach’s α > 0.70) [92]. The Kaiser-Meer-Olkin value was 0.96, above the cut-off value of 0.90. This result indicates that the data were suitable for factor analysis [52, 93, 94]. Thus, we applied confirmatory factor analysis to assess convergent validity and discriminative validity. Composite reliability (CR) and the average variance extracted (AVE) of the constructs are provided in Table 2. The CRs were greater than 0.70, and the AVEs were greater than 0.50, so the convergent validity of the scales was acceptable [95]. For complete statistics, consult Table 2. The correlations between each of the two constructs and the square root of average variance extracted (AVE) are shown in Table 3. For each construct, the square root of AVE was greater than the correlation coefficient between other constructs and itself, indicating an acceptable discriminant validity [96, 97].

**Table 2:** Measurement model for reflective measures

Constructs	M	SD	Loadings	alpha	CR	AVE
Performance Expectancy (PE)	3.76	0.58		0.85	0.85	0.53
PE1	3.74	0.71	0.53			
PE2	3.67	0.73	0.60			
PE3	3.78	0.78	0.69			
PE4	3.70	0.76	0.66			
PE5	3.89	0.70	0.65			
Effort Expectancy (EE)	3.91	0.71		0.85	0.84	0.64
EE1	3.88	0.83	0.76			
EE2	3.87	0.80	0.83			
EE3	3.90	0.83	0.78			
EE4	3.85	0.80	0.82			
Social Influence (SI)	3.77	0.72		0.83	0.83	0.62
SI1	3.74	0.89	0.74			
SI2	3.73	0.83	0.77			
SI3	3.84	0.78	0.68			
Facilitating Conditions (FC)	4.02	0.61		0.84	0.89	0.66
FC1	4.11	0.71	0.80			
FC2	4.02	0.67	0.80			
FC3	3.92	0.72	0.71			
FC4	3.96	0.64	0.76			
Perceived Risk (PR)	2.76	0.74		0.83	0.83	0.55
PR1	2.72	0.86	0.69			
PR2	2.92	0.94	0.77			
PR3	2.50	0.93	0.62			
PR4	2.90	0.91	0.74			
Price Value (PV)	3.65	0.69		0.78	0.79	0.56
PV1	3.54	0.73	0.69			
PV2	3.63	0.80	0.71			
PV3	3.57	0.87	0.77			
eHealth Literacy (EL)	3.58	0.64		0.89	0.89	0.57
EL1	3.66	0.69	0.61			
EL2	3.68	0.76	0.69			
EL3	3.67	0.76	0.72			
EL4	3.63	0.76	0.76			
EL5	3.76	0.78	0.69			
EL6	3.50	0.85	0.74			
EL7	3.62	0.72	0.72			
EL8	3.66	0.74	0.67			
Relation Quality (RQ)	3.55	0.67		0.92	0.92	0.69
RQ1	3.54	0.74	0.62			
RQ2	3.43	0.81	0.67			
RQ3	3.52	0.75	0.67			
RQ4	3.58	0.80	0.62			
RQ5	3.67	0.79	0.67			
Behavioral Intention (BI)	3.91	0.73		0.88	0.88	0.71
BI1	3.86	0.75	0.63			
BI2	3.91	0.84	0.66			
BI3	3.92	0.84	0.66			
Usage Behavior (UB)	3.66	0.78		0.90	0.90	0.70
UB1	3.58	0.89	0.64			
UB2	3.74	0.79	0.67			
UB3	3.53	0.79	0.65			
UB4	3.55	0.76	0.67			

*Note*: M: Mean; SD: standard deviations; Loadings: Factor loadings; alpha: Cronbach’s alpha; CR: Composite reliability; AVE: Average variance extracted

**Table 3:** Correlations matrix and square root of average variance extracted

	PE	EE	SI	FC	PR	PV	EL	RQ	UI	UB
PE	0.73									
EE	0.46	0.80								
SI	0.59	0.48	0.78							
FC	0.50	0.45	0.44	0.81						
PR	-0.45	-0.36	-0.41	-0.33	0.74					
PV	0.52	0.50	0.44	0.44	-0.43	0.75				
EL	0.61	0.60	0.50	0.47	-0.50	0.68	0.76			
RQ	0.67	0.45	0.57	0.40	-0.56	0.57	0.69	0.83		
BI	0.64	0.60	0.56	0.49	-0.43	0.68	0.75	0.66	0.84	
UB	0.65	0.53	0.52	0.46	-0.50	0.58	0.71	0.70	0.73	0.84

*Note*: PE: performance expectancy; EE: effort expectancy; SI: social influence; FC: facilitating conditions; PR: perceived risk; PV: price value; EL: eHealth literacy; RQ: relation quality; BI: behavioral intention; UB: usage behavior

We used *R*^2^ to test the explanatory capacity of our research model [98]. *R*^2^ of all constructs in our research model exceeded 0.10. In addition, the constructs in the research model accounted for more than 1.5% of the variance of the predicted structure, respectively. Therefore, our research model was adequate because it met the standard (*R*^2^ ≥ 0.10, predictor variables explaining ≥ 1.50% of variance) [99]. With regards to the *R*^2^ of the behavioral intention of the models, the integrated framework that combined UTAUT and patients’ information demands of OHCs (*R*^2^ = 0.70) outperformed the UTAUT model and other models which were commonly used in previous studies (*R*^2^ ≤ 0.52). It indicates that our research model can account for higher explained variance. Therefore, our research model has a stronger explanatory capacity than the best-known technology adoption models.

Next, we tested for the collinearity problem in our sample. Variance inflation factor (VIF) scores were used in this test. The results show that the VIF of all items in our sample were between 1.58 and 3.63, which meets the criteria (the value of VIF should be between 0.20 and 5.00) [100], indicating that there is no collinearity problem in our sample.

Next, we assessed model fit. According to the literature, we applied standardized root mean square residuals (SRMR), normed fit index (NFI), and root mean square error correlation (RMS_theta_) to test the model fit. According to the results, the SRMR value was 0.05, consistent with the suggested result (less than 0.08) [101], suggesting a satisfactory model fit. Furthermore, the NFI score was 0.96, greater than the advised value of 0.90 [102]. It revealed that the model fit was satisfactory. Moreover, the value of RMS_theta_ was 0.10 (less than 0.12), indicating that the model was well-fitting [103].

Finally, we used Harmon’s single-factor test to evaluate common technique bias [104]. The results indicated that the primary characteristic roots of the variables were larger than 1.00. The first component explained 35.68% of the variation, less than the critical value of 50.00% [105]. In addition, we included the common method variance component in our research model as a latent variable. The results revealed no statistically significant change in model fit between the model with the common method variance factor and the original model [105]. Above all, the common method variance does not exist in our study.

### Hypothesis testing

We used SmartPLS 3.3.2 software to analyze the important relationship between the variables and verify the hypothesis. In previous studies, age, gender, experience, and education level were used as control variables [47, 106, 107]. In order to consider the potential influence of these control variables, we added them to the research model. The effects of the control variables (age, gender, experience, and education level) were assessed by Cohen ƒ^2^ [108]. As shown in Table 4, ƒ^2^ values were all between 0.002 and 0.003. Therefore, the effects of the control variables are limited (insignificant: ƒ^2^$<$0.020; small: 0.020$\le$ƒ^2^$<$0.150; medium: 0.150$\le$ƒ^2^$<$0.300; and large: ƒ^2^$\ge$0.350) [108].

**Table 4:** Test results for control variables

Variables	*R* ^2^		Control variable effect		
	With control variable	Without control variable	Δ*R*^2a^	*f* ^*2b*^	Effect
Behavioral intention	0.175	0.172	0.003	0.003	insignificant
Usage behavior	0.161	0.159	0.002	0.002	insignificant

*Note*: ^a^Δ*R*^2^: *R*^2^_with control variables_ – *R*^2^_without control variables_; ^b^ƒ^2^: Cohen ƒ^2^

**Fig. 2:**
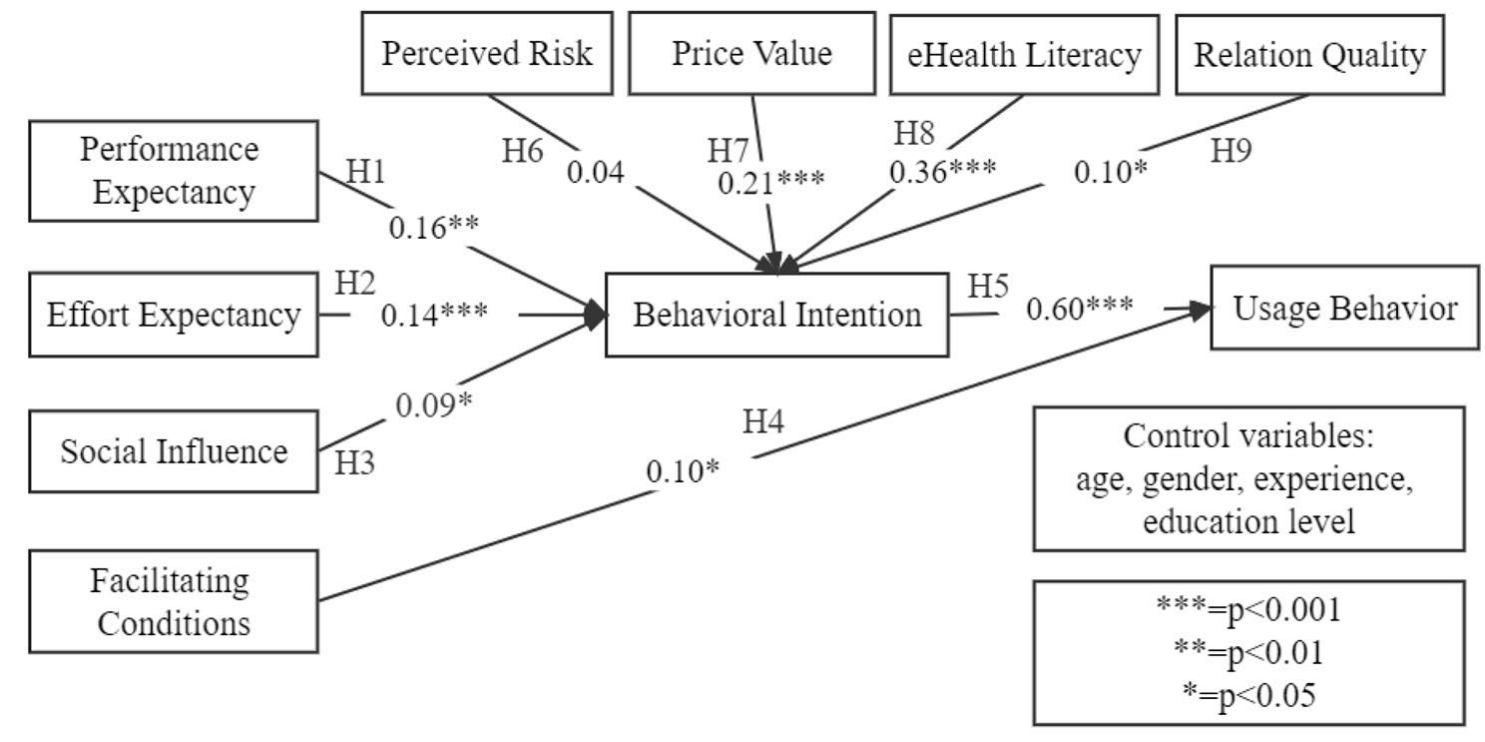
Research model with PLS-SEM results

The PLS-SEM results of the research model are illustrated in Fig. 2, and Table 5 shows the magnitude and significance of the path coefficients and the hypothesis testing results. The results reveal that performance expectancy, effort expectancy, social influence, price value, eHealth literacy, and relation quality all positively affect behavioral intention. Among them, price value and eHealth literacy have larger path coefficients than other factors, indicating that these two factors strongly impact behavioral intention. In addition, facilitating conditions and behavioral intention have positive impact on usage behavior. Therefore, H1, H2, H3, H4, H5, H7, H8 and H9 are supported. However, H6 is not supported at the p > 0.05 level, that is, the impact of perceived risk on behavioral intention is not significant.

**Table 5:** Hypothesis testing results

Hypothesis	Path Coefficient	*t*-value	*p*-value	Significance	Supported
H1:PE→BI	0.16	2.99	0.00**	Significant	Yes
H2: EE→BI	0.14	3.76	0.00***	Significant	Yes
H3: SI→BI	0.09	1.97	0.05*	Significant	Yes
H4:FC→UB	0.10	2.21	0.03*	Significant	Yes
H5:BI→UB	0.60	11.65	0.00***	Significant	Yes
H6: PR→BI	0.04	1.04	0.30	Not Significant	No
H7: PV→BI	0.21	4.86	0.00***	Significant	Yes
H8: EL→BI	0.36	5.89	0.00***	Significant	Yes
H9: RQ→BI	0.10	1.98	0.05*	Significant	Yes

***=p < 0.001.

**=p < 0.01.

*=p < 0.05.

## Discussion

### Main findings

First of all, the results indicate that patients’ behavioral intention has a significant positive impact on usage behavior, and the degree of this influence is large, exceeding 50.00% (path coefficients = 0.60). This finding is in accordance with previous studies that elucidated the significant positive relationship between behavioral intention and usage behavior in IT (Information Technology) adoption contexts [27, 55, 109]. Additionally, facilitating conditions also have a significant positive effect on usage behavior, which is supported by previous studies clarifying that facilitating conditions significantly contribute to usage behavior [110, 111]. Thus, we believe that patients’ attitudes toward OHCs and ease of use are significant factors influencing their adoption of OHCs.

Second, performance expectancy, effort expectancy, and social influence have significant positive impacts on patients’ behavioral intention of using OHCs. The UTAUT model suggests that the above three variables are important determinants of behavioral intention. Therefore, these findings are consistent with the conventional findings of the UTAUT model [13, 20, 25]. In particular, performance expectancy had the greatest effect on behavioral intention (path coefficients = 0.16), indicating that when deciding on using OHCs, patients are most concerned about whether they can get the information and services they want to receive. The findings demonstrate that patients’ behavioral intention to use OHCs largely depends on their perception of the help that OHCs can bring to them and the difficulty of using OHCs. If patients think that OHCs can significantly improve the efficiency of getting diagnosis and increase the speed of information acquisition, or they believe that technical problems can be solved in time during the use of OHCs, they will be willing to use OHCs. In addition, patients are likely to be influenced by their friends and relatives. If others have positive comments on OHCs, patients will trust on OHCs, thereby improving their intention to use OHCs.

Third, the results reveal that perceived risk has an insignificant impact on patients’ behavioral intention of using OHCs. Interestingly, our finding differs from previous studies’ results as these studies found a statistically significant effect of perceived risk on behavioral intention [40, 62, 112]. The reasons are as follows. First of all, patients mainly use the free modules of OHCs, such as the problem posts and health information modules. Therefore, they may not be sensitive to the economy and privacy. In addition, for patients, the information obtained from OHCs is considered a reference rather than psychological dependence. Finally, patients may be inured to the existence of risk in the context of the Internet environment.

Fourth, price value has a significant positive influence on patients’ behavioral intention of using OHCs, which means that price value plays an important role in influencing patients’ intention to use OHCs. This result is consistent with the findings of previous studies [13, 113]. The finding indicated that patients would weigh whether their expenditures on OHCs are worth it. Setting a reasonable price is a sufficient condition for OHCs to attract users.

Fifth, consistent with the conclusions inferred from the previous literature [74, 76, 114], eHealth literacy has a significant positive effect on behavioral intention. More importantly, eHealth literacy is the most important determinant of patients’ behavioral intention to use OHCs. eHealth literacy reflects the ability of users to access and process health information. Patients with strong abilities in information reception, information processing, information understanding, and information discrimination are more adept at utilizing the information provided on OHCs to help themselves, thereby increasing their willingness and behavior to use OHCs. The results suggest that patients’ own perceptions and ability to adopt health information can have a significant impact on their intention to use OHCs. Therefore, enhancing patients’ positive attitudes toward OHCs can also start from the perspective of patients’ understanding and abilities of medical and health knowledge.

Last, the empirical result indicates that relation quality has a significant positive effect on patients’ behavioral intention of using OHCs. This finding is consistent with prior studies indicating that users with high relation quality are more willing to use the corresponding information technology [44]. This finding suggests the importance of patients’ trust and satisfaction with the platform has been highlighted. The relationship between patients and the platform needs to be paid attention to.

### Theoretical implications

This study contributes to OHCs literature by constructing a comprehensive model for explaining patients’ adoption behavior of OHCs based on the UTAUT model and the analysis results of patients’ information demands in OHCs. First, this study enriches the literature on the adoption of OHCs. Previous literature lacked an understanding of the role of patients’ abilities to identify and process online health information as well as patient-platform relationships. Our findings enrich the knowledge of the impact of eHealth literacy and relation quality with OHCs and provide new insights from the perspective of patient’s intrinsic motivation to enhance the understanding of the factors to be considered in technology adoption models in the context of OHCs.

Second, this study extends UTAUT in OHCs and specific users (patients), enriching the literature on UTAUT application scenarios. This study proposes a more comprehensive and appropriate model to understand patients’ motivations to use OHCs by extending the UTAUT model based on the actual information demands of patients in OHCs. The extended UTAUT model proposed in this study enhances the understanding of the adoption of OHCs. The findings of the main hypotheses presented in the research model of this study are found to be consistent with the UTAUT model results, providing further support for the application of UTAUT to OHCs. In addition, the findings of this study enrich the literature of UTAUT by explaining the factors specific to the context of this study that influence the adoption of OHCs, and the context-based insights are well regarded as complementary to the existing knowledge in the field of technology adoption.

Third, our study extends the scope of application of the findings. Given that most related studies have examined OHCs in developed countries, the absence of study in other regions of the world, where the majority of the population resides, makes concluding other countries problematic. Our research model is validated by the data collected in China, which currently has the largest population. This study contributes to the literature on the development of OHCs in China and other developing countries with similar digital health technology development to China.

### Practical implications

First of all, the findings showed that patients’ behavioral intention and facilitating conditions have significant positive impacts on usage behavior. Therefore, policymakers can promote the popularity of OHCs by improving patients’ intention to use OHCs, making OHCs compatible, and setting low usage conditions for the patients. For example, it may be beneficial for policymakers to collaborate with healthcare providers and other stakeholders to develop educational campaigns that promote the benefits of using OHCs, and to provide training and support for patients.

Second, the results indicated that high performance expectancy, effort expectancy, and social influence could significantly improve patients’ positive attitudes toward OHCs. The findings inform the operators of OHCs that they need to focus on information quality, friendly interfaces, obvious tags, and web navigation of OHCs to enhance the interaction between OHCs and the users. In addition, the government and hospitals can increase the promotion and publicity of OHCs and pay attention to patients’ evaluations to gain a good word-of-mouth reputation in order to attract more users.

Third, price value has a positive impact on patients’ behavioral intention of using OHCs. Therefore, the operators of OHCs should set a price based on market research that matches the service and information quality provided by OHCs. They also need to understand the actual attitude of patients towards information quality so as to try to narrow the gap between perceived information quality and actual information quality.

Fourth, eHealth literacy is an important factor influencing patients’ usage intention of OHCs. Therefore, it is a good choice for the government to strengthen the promotion of basic medical and health knowledge, healthy lifestyles and behaviors, and basic skills in obtaining information and services online. Besides, the hospitals can try to encourage the public to strengthen relevant learning in order to improve their ability to search, understand, evaluate, and use the information. Physicians would better explain health-related knowledge to patients actively, correct patients’ misconceptions, and guide patients to identify and use the information correctly.

Finally, the results show that relation quality has a considerable favorable influence on patients’ behavioral intention to use OHCs. Therefore, the operators of OHCs should make efforts to ensure the authority and professionalism of the hospitals and physicians in OHCs and enhance the quality of information and services in it. They can cooperate with reliable, authoritative, and professional hospitals and physicians, supervise the information released in OHCs, and strictly handle patients’ complaints.

### Limitations and future research

It should be noted that this study has some limitations. First, this study used a cross-sectional investigation without dynamically studying the changes in participants’ attitudes toward all variables. In the subsequent research, longitudinal study methods will be adopted to test research models. Besides, the statistics on the patients of OHCs may change with the rapid growth of OHCs. Most of the patients surveyed are young and highly educated groups. Other groups may be expanded in the future, and we will collect the data again to verify the model at that time. Last, there are differences in the development of digital health technologies in developing countries. Future research will be conducted in more developing countries with different levels of digital health development to form a comprehensive study that takes into account the impact of technology developments specific to other countries.

## Conclusion

In this study, we introduce a comprehensive research model by extending the UTAUT model based on patients’ information demands of OHCs. Alongside the impact of the constructs of UTAUT (i.e., performance expectancy, effort expectancy, and social influence have significant positive impacts on the behavioral intention of using OHCs, while behavioral intention and facilitating conditions have significant positive impacts on the usage behavior), this study identifies the significant positive effects of price value, eHealth literacy, and relation quality on behavioral intention of using OHCs. These findings suggest that the operators of OHCs need to focus on improving the construction of the platform, including compatibility and information content. Also, the operators of OHCs should increase the publicity of OHCs, set reasonable prices, publicize health knowledge, and protect patients’ property and information security. The hospitals and physicians can also provide patients with courses that teach them how to judge, understand and use the information in OHCs appropriately. The findings expand the understanding of the usage of OHCs and contribute to both the theories of adopting information technology and practical applications that promote the spread of OHCs.

## Electronic supplementary material

Below is the link to the electronic supplementary material.

Supplementary Material 1

## Data Availability

The datasets used and/or analysed during the current study are available from the corresponding author on reasonable request.

## List of Abbreviations

OHCs: Online health communities
