# Supplementary material for: What drives the adoption of online health communities? An empirical study from patient-centric perspective
Source: BMC Health Serv Res. 2023 May 23;23:524. doi: 10.1186/s12913-023-09469-6 (PMC10204683; doi:10.1186/s12913-023-09469-6)
Supplement: Supplementary file 1 — Supplementary Material 1 [file 12913_2023_9469_MOESM1_ESM.docx]

**Appendix** Measurement instruments.

| **Construct** | **Items** | **Source** |
| --- | --- | --- |
| Performance Expectancy | 1. I think online health communities would provide me with medical information and services timely. | Venkatesh et al. [20] |
|  | 1. I think online health communities would provide me with valuable and accurate information resources. |  |
|  | 1. I think using online health communities would make it easier for me to obtain medical services. |  |
|  | 1. I think using online health communities would improve the quality of my life. |  |
|  | 1. I think online health communities are useful in my daily life. |  |
| Effort Expectancy | 1. I think it is easy to learn to use online health communities. | Venkatesh et al. [20] |
|  | 1. I think online health communities are easy to operate. |  |
|  | 1. I think it is easy to visit online health communities skillfully. |  |
|  | 1. I think it is easy to search information and services in online health communities. |  |
| Social Influence | 1. I will feel uneasy if my friends use online health communities but I do not. | Venkatesh et al. [20] |
|  | 1. People who influence my behavior think that I should use online health communities. |  |
|  | 1. People who are important to me think that I should use online health communities. |  |
| Facilitating Conditions | 1. I have the resources (equipment, network, etc.) to support my use of online health communities. | Venkatesh et al. [20] |
|  | 1. My knowledge can support my use of online health communities. |  |
|  | 1. The platforms of online health communities are compatible with other software I use. |  |
|  | 1. I can ask for help online or from others when I have problems in the process of using online health communities. |  |
| Perceived Risk | 1. I am concerned that there is a payment risk in the online health communities, which cannot guarantee the security of my property. | Sun and Lu [27] |
|  | 1. I’m worried about personal privacy exposure on healthcare websites. |  |
|  | 1. I’m worried about wasting time to use online health communities. |  |
|  | 1. I’m worried about that some functions of online health communities are still imperfect. |  |
| Price Value | 1. I think online health communities is reasonably priced. | Yuan et al. [67] |
|  | 1. I think the services of online health communities are valuable for the price. |  |
|  | 1. At the current price, online health communities provide a good value. |  |
| eHealth Literacy | 1. I know how to find useful resources through online health communities. | Norman and Skinner [73] |
|  | 1. I know how to solve my health-related problems through online health communities. |  |
|  | 1. I know which resources are available in online health communities. |  |
|  | 1. I know where can get useful resources in OHCs. |  |
|  | 1. I know how to help myself with the information provided in online health communities. |  |
|  | 1. I know what skills required to evaluate the medical and health resources in OHCs. |  |
|  | 1. I can distinguish between high-quality and low-quality information in online health communities. |  |
|  | 1. I am confident in making good decisions with using the information provided by online health communities. |  |
| Relation Quality | 1. I think the information in online health communities is reliable. | Chen et al. [43] |
|  | 1. I think that the hospitals, physicians and medicines in online health communities are authoritative, credible and authentic. |  |
|  | 1. I think the services provided in online health communities are reliable. |  |
|  | 1. I had good overall experience with using online health communities. |  |
|  | 1. I think the decision to use online health communities is right. |  |
| Behavioral Intention | 1. I think I will be willing to learn to use online health communities. | Venkatesh et al. [20] |
|  | 1. I think I will be willing to try to use online health communities in the future. |  |
|  | 1. I am willing to recommend online health communities to others if the experience is good. |  |
| Usage Behavior | 1. Recently, I plan to use online health communities. | Venkatesh et al. [20], Sun and Lu [27] |
|  | 1. I will continue to use online health communities in the future. |  |
|  | 1. I am willing to consult about health issues through online health communities. |  |
|  | 1. I am willing to obtain medical and health information through online health communities. |  |
